# Insight into the emerging role of SARS-CoV-2 nonstructural and accessory proteins in modulation of multiple mechanisms of host innate defense

**DOI:** 10.17305/bjbms.2020.5543

**Published:** 2021-10

**Authors:** Abualgasim Elgaili Abdalla, Jianping Xie, Kashaf Junaid, Sonia Younas, Tilal Elsaman, Khalid Omer Abdalla Abosalif, Ayman Ali Mohammed Alameen, Mahjoob Osman Mahjoob, Mohammed Yagoub Mohammed Elamir, Hasan Ejaz

**Affiliations:** 1 Department of Clinical Laboratory Sciences, College of Applied Medical Sciences, Jouf University, Al Jouf, Saudi Arabia; 2 Department of Medical Microbiology, Faculty of Medical Laboratory Sciences, Omdurman Islamic University, Omdurman, Sudan; 3 Institute of Modern Biopharmaceuticals, State Key Laboratory Breeding Base of Eco-Environment and Bio-Resource of the Three Gorges Area, Key Laboratory of Eco-environments in Three Gorges Reservoir Region, Ministry of Education, School of Life Sciences, Southwest University, Beibei, Chongqing, China; 4 Department of Pathology, Tehsil Headquarter Hospital Kamoke, District Gujranwala, Kamoke, Pakistan; 5 Department of Pharmaceutical Chemistry, College of Pharmacy, Jouf University, Al Jouf, Saudi Arabia; 6 Department of Pharmaceutical Chemistry, Faculty of Pharmacy, Omdurman Islamic University, Omdurman, Sudan; 7 Department of Chemical Pathology, Faculty of Medical Laboratory Sciences, University of Khartoum, Khartoum, Sudan

**Keywords:** COVID-19, SARS-CoV-2, nonstructural proteins, accessory proteins, proinflammatory, interferon, immune evasion, immunomodulators

## Abstract

Coronavirus disease-19 (COVID-19) is an extremely infectious disease caused by severe acute respiratory syndrome coronavirus 2 (SARS-CoV-2) that has become a major global health concern. The induction of a coordinated immune response is crucial to the elimination of any pathogenic infection. However, SARS-CoV-2 can modulate the host immune system to favor viral adaptation and persistence within the host. The virus can counteract type I interferon (IFN-I) production, attenuating IFN-I signaling pathway activation and disrupting antigen presentation. Simultaneously, SARS-CoV-2 infection can enhance apoptosis and the production of inflammatory mediators, which ultimately results in increased disease severity. SARS-CoV-2 produces an array of effector molecules, including nonstructural proteins (NSPs) and open-reading frames (ORFs) accessory proteins. We describe the complex molecular interplay of SARS-CoV-2 NSPs and accessory proteins with the host’s signaling mediating immune evasion in the current review. In addition, the crucial role played by immunomodulation therapy to address immune evasion is discussed. Thus, the current review can provide new directions for the development of vaccines and specific therapies.

## INTRODUCTION

Severe acute respiratory syndrome coronavirus 2 (SARS-CoV-2) is a newly emergent member of the *Coronaviridae* family and belongs to the *Betacoronavirus* genus and *Sarbecoronavirus* subgenus [1-3]. SARS-CoV-2 consists of a single-stranded, linear, and non-segmented positive-sense RNA core encased within a helical capsid and encompassed by a lipid envelope [4]. The SARS-CoV-2 RNA genome is roughly 29.89 kb in size and shares 82% and 50% nucleotide sequence identity with the severe acute respiratory syndrome coronavirus (SARS-CoV) and Middle East respiratory syndrome coronavirus (MERS-CoV), respectively [4].

SARS-CoV-2 causes coronavirus disease-19 (COVID-19) is the most widespread pandemic disease of the 21st century. As of March 1, 2021, it has affected over 113 million people and has been responsible for more than 2.5 million deaths globally [5]. The presentation of COVID-19 can range from subclinical, mild symptoms, including fever, fatigue, and cough, to life-threatening symptoms, such as dyspnea and acute respiratory distress syndrome (ARDS) [6-8]. The pathophysiology of COVID-19 depends on the virus’s ability to manipulate the host immune responses [9,10]. SARS-CoV-2 can modulate the host immune system in its favor by blocking antiviral immunity and promoting tremendous inflammatory reactions that have been associated with illness severity [11,12]. Therefore, understanding the mechanisms through which SARS-CoV-2 commandeers the immune response will improve current efforts toward drug design and development.

Two-thirds of the SARS-CoV-2 genome encodes nonstructural proteins that are required for viral RNA transcription and translation [13,14]. Several other open-reading frames (ORFs) accessory proteins that are not necessary for viral replication but contribute to immune evasion and pathogenesis [15]. The current review describes the current state of knowledge regarding how the SARS-CoV-2 nonstructural and accessory proteins mediate the hijacking of the host immune response.

### Immune response dysregulation in COVID-19 patients

SARS-CoV-2 is a distinct respiratory pathogen that has developed several strategies to evade the immune response, allowing the virus to remain and replicate in human respiratory tissue. SARS-CoV-2 can cause a severe deficiency in type I interferon (IFN-I) production and activity, which has been significantly associated with increased viral load, inflammatory reactions, and disease severity [16]. COVID-19 patients present with the significantly impaired and delayed secretion of IFN-I and IFN-III compared with flu patients. High levels of IFN-III reduce viral loads and hasten the clearance of infection, and higher concentrations of IFN-III relative to the concentrations of IFN-I can relieve critical illness in COVID-19 patients. Proinflammatory cytokines, such as tumor necrosis factor-alpha (TNF-α), interleukin-6 (IL-6), IL-1, and IL-8, have been significantly associated with severe COVID-19 cases [17,18]. Surprisingly, increased levels of IFN-I have been directly linked to disease progression and acute respiratory injury [16,18,19].

SARS-CoV-2 infection promotes apoptosis, which can augment the acute inflammatory reaction and compromise the lymphocytic response. High levels of apoptotic lung cells and inflammatory cell infiltration were observed in the lung sections collected from postmortem COVID-19 cases [20]. SARS-CoV-2 can induce the apoptosis of pneumocytes and endothelial cells, resulting in tremendous levels of lung destruction [17]. Several pro-apoptotic genes were found to be significantly upregulated in peripheral blood mononuclear cells (PBMCs) derived from COVID-19 patients with reduced lymphocyte counts, which suggests a potential role for apoptosis in lymphocytopenia among COVID-19 patients [21]. The levels of apoptosis mediator proteins, such as caspase-8 and TNF superfamily member 14 (TNFSF14), were significantly higher in COVID-19 patients than those in healthy control [22].

SARS-CoV-2 can also manipulate both the cellular and humoral immune responses. In severe COVID-19 cases, delayed virus elimination was significantly correlated with an impaired antigenic presentation and the severe dysfunction of cytotoxic T lymphocytes (CTL), natural killer (NK) cells, and B lymphocytes (B cells) [23]. In addition, critically ill COVID-19 individuals showed a significant decrease in CTL and CD4^+^ helper T cells, accompanied by an increased neutrophil count [17,24]. Thus, a clear and thorough understanding of the molecular interplay that occurs between SARS-CoV-2 and the immune system can inform the design and development of better therapeutic interventions.

### Immunomodulatory SARS-CoV-2 nonstructural proteins

SARS-CoV-2 encodes 16 NSPs, designated NSP-1 through NSP-16, which are necessary for viral replication [14]. The interactions between some of these SARS-CoV-2 NSPs and components of host cell signaling pathways for the manipulation of defense mechanisms have been explored.

**FIGURE 1:**
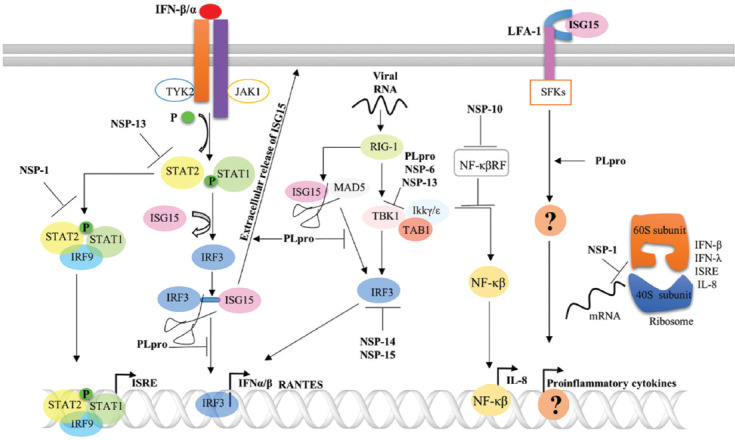
Cross-talk between SARS-CoV-2 nonstructural proteins (NSPs) and host cell signaling pathways mediates immune modulation. SARS-CoV-2 NSP-1, PLpro, and NSP-13 interfere with type I IFN signaling by mediating the inhibition of STAT1 and STAT2 phosphorylation, the deISGylation of IRF3, and the suppression of STAT2 phosphorylation, respectively. SARS-CoV-2 PLpro, NSP-6, and NSP-13 reduce TBK1 (TANK-binding kinase) phosphorylation, leading to the blockade of the RIG-1 pathway’. PLpro can also interfere with RIG-1 signaling through the deISGylate of MAD5 (melanoma differentiation-associated protein 5). NSP-14 and NSP-15 abolish IRF3 nuclear translocation, leading to the inhibition of type I IFN expression. SARS-CoV-2 PLpro and NSP-10 can enhance the proinflammatory response, and PLpro can enhance the extracellular release of ISG, which can trigger proinflammatory mediator production following binding with the LFA-1 receptor. NSP-10 can block NF-κB repression, promoting NF-κB pathway activation and IL-8 production. NSP-1 can suppress IFN-β, IFN-λ, ISRE, and IL-8 secretion by blocking mRNA translation.

NSP-1, expressed by *Betacoronavirus* species, can negatively regulate the biosynthesis of host proteins by mediating the post-transcriptional degradation of mRNAs [25,26]. However, the SARS-CoV-2 NSP-1 was shown to blocks host mRNA translation by binding firmly to the mRNA entry channel in the 40s ribosome rather than inducing mRNA degradation [27]. The expression of SARS-CoV-2 NSP-1 in human embryonic kidney-derived HEK293T cells potently abolished IFN-I, IFN-III, and IL-8 secretion upon challenge with Sendai virus, which is a retinoic acid-inducible gene-1 (RIG1) signaling agonist. The SARS-CoV-2 NSP-1 can also hijack the IFN-mediated transcription of the IFN-stimulated response element (ISRE), which is crucial for the expression of IFN-stimulated genes (Figure 1). SARS-CoV-2 NSP-1 has been shown to target 18 S rRNA in the 40s ribosome, leading to the complete blockage of host mRNAs translation and the inhibition of type I IFN signaling [28]. NSP-1 can also disrupt type I IFN signal transduction by inhibiting the phosphorylation and nuclear translocation of signal transducer and activator of transcription (STAT1) and STAT2 (Figure 1) [29]. SARS-CoV-2 appears to be a more potent antagonizer of type I IFN signaling than either SARS-CoV or MERS-CoV [29].

Papain-like protease (PLpro) is an NSP-3 domain with deubiquitinating and deISGylating activities that target components of the host’s innate immune signaling pathways. SARS-CoV PLpro consistently suppresses the antiviral innate immune response by antagonizing IFN regulatory factor 3 (IRF3) and the nuclear factor kappa B (NF-κB) signaling pathways [30,31]. SARS-CoV-2 and SARS-CoV PLpro share approximately 83% amino acid homology and feature conserved ubiquitin-1 (Ub-1)-binding residues. However, they feature differing Ub-2-binding residues, which results in a decrease in the deubiquitinating activity of SARS-CoV-2 PLpro relative to that of SARS-CoV PLpro. The ectopic expression of SARS-CoV-2 PLpro in HEK293T cells weakly suppress type I IFN production upon treatment with a RIG-1 agonist compared with the activity observed for SARS-CoV PLpro [32]. However, Shin et al. revealed that the expression of SARS-CoV-2 PLpro in HeLa cells suppressed the type I IFN response by directly cleaving ISG15 from IRF3, thereby blocking IRF3 nuclear translocation (Figure 1). The expression of PLpro can reduce the phosphorylation of TANK-binding kinase (TBK-1), which results in the suppression of both the IRF3 and NF-κB pathways. The inhibition of PLpro activity can reduce SARS-CoV-2 replication and the induction of cytopathic effects [33]. Furthermore, SARS-CoV-2 PLpro can deISGylate melanoma differentiation-associated protein 5 (MAD5), resulting in a significant reduction in the transcription levels of IFN-β and regulated upon activation, normal T lymphocyte expressed and secreted (RANTES) [34]. SARS-CoV-2 PLpro can also promote the extracellular release of ISG15 when co-expressed with a plasmid expressing FLAG-ISG15 in HEK293 T cells. Extracellular ISG15 can bind to the integrin lymphocyte function-associated antigen-1 (LFA-1), resulting in IFN-γ production by natural killer 92 (NK-92) cells [35]. The recombinant ISG15-mediated stimulation of PBMCs from healthy donors results in the upregulation of proinflammatory cytokine secretion, particularly IL-1, IL-6, IFN-γ, and TNF-α, in addition to several chemokines [36]. These findings suggested that PLpro is a SARS-CoV-2 virulence factor that can inhibit the antiviral innate immune response and promote proinflammatory cytokine production (Figure 1).

SARS-CoV-2 NSP-6 is a vacuolar ATPase that can interact with the host sigma factor, leading to the modulation of the endoplasmic reticulum (ER) stress response [37]. NSP-6 antagonizes type I IFN production by blocking IRF3 activation. The overexpression of NSP-6 in HEK293T cells can suppress RIG-1 signal-mediated IRF3 phosphorylation by targeting TBK-1 (Figure 1) [29].

SARS-CoV-2 NSP-10 is a master regulator of RNA repair and viral mRNA capping activity through the promotion of the 3-exoribonuclease and methyltransferase activities of NSP-14 and NSP-16, respectively [38]. In the study by Li et al., NSP-10 could mediate IL-8 activation by targeting NF-κB repressing factor (NF-κBRF; Figure 1) [39]. IL-8 is a crucial inflammatory mediator that can enhance the recruitment of polymorphonuclear (PMN) leukocytes to the site of infection, which can enhance disease severity by promoting inflammatory reactions and lung epithelial cell destruction [40]. SARS-CoV-2 NSP-13 is an enzyme with RNA helicase and nucleoside triphosphate hydrolase (NTPase) activities [41]. NSP-13 plays a decisive role in SARS-CoV-2 pathogenesis by manipulating type I IFN production and signaling. The ectopic expression of NSP-13 in HEK293T cells was able to suppress RIG-1-mediated IFN-β expression by inhibiting the nuclear translocation of IRF3 [32]. NSP-13 can abolish the phosphorylation of TBK-1, which can directly affect IRF3 activation (Figure 1). NSP-13 can also interfere with IFN-α/β downstream signaling by preventing STAT2 phosphorylation [29].

SARS-CoV-2 NSP-14 and NSP-15 are an exoribonuclease and endoribonuclease, respectively. The expression of NSP-14 or NSP-15 in HEK293T cells can downregulate IFN-β transcription by inhibiting the nuclear translocation of IRF3 following activation of RIG-1 signaling (Figure 1) [32]; however, the mechanisms through which NSP-14 and NSP-15 inhibit IRF3 nuclear translocation remains unclear.

### Immunomodulatory SARS-CoV-2 accessory proteins

Nine ORFs accessory proteins including ORF3a/b, ORF6, ORF7a/b, ORF8, ORF9b/c, and ORF10, are predicted to be encoded by the SARS-CoV-2 genome [13]. These proteins are unnecessary for viral replication and vary in number and sequence from those encoded by SARS-CoV [15], which may explain the uniquely enhanced virulence and pathogenesis of SARS-CoV-2. SARS-CoV-2 ORFs are thought to play a master role in pathogenesis by negatively regulating the immune response.

**FIGURE 2:**
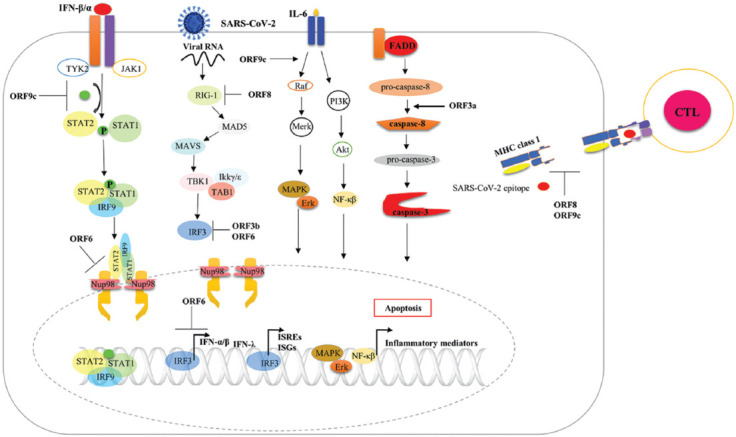
Mechanisms through which SARS-CoV-2 accessory proteins hijack the immune system. SARS-CoV-2 ORF6 can prevent type I IFN signaling pathway by blocking nuclear import system. ORF9c can also abrogate type I IFN response by downregulating several IFN pathway components. SARS-CoV-2 ORF3b, ORF6, and ORF8 can block the RIG-1 signaling-mediated expression of type I IFN, ISREs, and ISGs. ORF3b and ORF6 inhibit IRF3 translocation to the nucleus. SARS-CoV-2 ORF9c can promote the expression and translation of proinflammatory mediators, such as IL-6, MAPK, and NF-κB. SARS-CoV-2 ORF3b can enhance the apoptosis process through the activation of caspase-8. SARS-CoV-2 ORF8 and ORF9c can interfere with the antigenic presentation process through the downregulation of MHC class I expression.

ORF3a is an ion channel protein with a high binding affinity for chloride ions [42]. The ectopic expression of SARS-CoV-2 ORF3a can significantly promote apoptosis in multiple cell types, including HEK293T, HepG2, and VeroE6 cells. ORF3a can trigger the extrinsic apoptotic pathway by promoting the cleavage and activation of caspase-8 (Figure 2) [43]. *In vivo* study performed in human angiotensin-converting enzyme 2 (hACE2) transgenic mice demonstrated that SARS-CoV-2 infections could induce caspase-8 activation, leading to lung epithelial cell apoptosis and the increased secretion of proinflammatory cytokines [20]. Therefore, the role played by SARS-CoV-2 ORF3a in the activation of the inflammatory process needs to be addressed.

SARS-CoV-2 ORF3b is an immunodominant protein that has been shown to induce high levels of antibody production during COVID-19 infections [44]. The gene encoding ORF3b has high variability, ranging from a short ORF with a premature stop codon [13] to the loss of the premature stop codon [45], and the complete loss, which has been observed in the circulating SARS-CoV-2 strains identified in several countries [46]. The expression of the short SARS-CoV-2 ORF3b protein in a human lung epithelial (A549) cell line was shown to be more efficient for the inhibition of type I IFN production following Sendai virus infection than that encoded by SARS-CoV (Figure 2). Interestingly, the long SARS-CoV-2 ORF3b protein variant was found more potently block the type I IFN production than the shorter variant. The SARS-CoV-2 strain possesses an elongated ORF3b variant that has been associated with more severe COVID-19 illness [45]. Further experiments remain necessary to determine how SARS-CoV-2 ORF3b blocks IRF3 nuclear translocation.

ORF6 has been found to be expressed in human lung epithelial cells, suggesting its potential role in the cross-talk between SARS-CoV-2 and host signaling pathways [47]. Consistently, HEK293T cells that express SARS-CoV-2 ORF6 exhibit significantly lower transcription levels of IFN-β, ISREs, IFN-stimulated genes (ISGs), and NF-κB upon exposure to Sendai virus infection or recombinant IFN-β compared with cells transfected with a control vector (Figure 2) [48]. SARS-CoV-2 ORF6 can also strongly suppress IFN-I and IFN-III transcription and translation in HEK293 T cells in response to a RIG-1-inducer or Sendai virus (Figure 2) at levels comparable to those mediated by SARS-CoV ORF6 [32]. Moreover, the ectopic expression of SARS-CoV-2 ORF6 in HEK293 T cells can prevent RIG-1, MAD5, mitochondrial antiviral signaling protein (MAVS), and IRF3-mediated activation of IFN-β expression. SARS-CoV-2 ORF6 has also been shown to inhibit the expression of antiviral immune mediators by blocking the nuclear translocation of IRF3 and STAT1 leading to antagonize IFN-I signaling [49], which a recent study suggested was directly mediated by the interaction between SARS-CoV-2 ORF6 and the nuclear importer karyopherin-a 2 protein (KPNA2) [29]. SARS-CoV-2 ORF6 also appears to disrupt the nuclear import system by directly targeting the nuclear pore complex to suppress STAT1 nuclear localization (Figure 2), interacting with nucleoporin 98 (Nup98) at the nuclear membrane, preventing the interactions between Nup98 and KPNA1 and 2 [50].

SARS-CoV-2 ORF8 is a secreted protein that can be detected in the cultured supernatant of SARS-CoV-2-infected A549 and HEK293T cells and in the serum of COVID-19 patients [51]. High levels of ORF8 expression and secretion have been reported in a SARS-CoV-2 *in vitro* infection model in Vero CCL-18 cells [52], suggesting a potential role for ORF8 in the immune evasion process to promote viral growth. The ectopic expression of SARS-CoV-2 ORF8 in HEK293T cells can prevent IFN-β and RIG-1 pathway-mediated ISRE, ISG, and NF-κB transcription (Figure 2) [48]. ORF8 can also reduce the CTL killing capacity by interfering with the viral antigenic presentation. The ectopic expression of ORF8 in HEK293T cells was shown to downregulate the expression of major histocompatibility (MHC) class I molecule (Figure 2) [52,53].

SARS-CoV-2 ORF9c is a small membrane-anchored protein that plays an indispensable role in the suppression of the host immune response. The overexpression of ORF9c in A549 cells induces an imbalance in the immune response by downregulating signaling-mediated antigenic presentation, and the type I IFN response. It upregulates the expression of proinflammatory mediators, such as IL-6, mitogen-activated protein kinase (MAPK), and components of the NF-κB pathway (Figure 2) [54]. An in-depth study remains necessary to explore the underlying mechanisms through which SARS-CoV-2 ORF9c affects innate immunity.

### Immunomodulation as an attractive therapeutic option for COVID-19

The deleterious consequences associated with COVID-19 have placed considerable strain on global healthcare systems. Accordingly, extensive studies have been performed to explore the vast range of potential therapeutic approaches and modalities to address COVID-19, although no single drug has yet been identified as efficacious for the clinical management of COVID-19 patients [55]. The attempted approaches can conveniently be classified as follows: (i) the exploration of known antiviral drugs, such as IFNs; (ii) the screening of existing compound libraries for those with potential inhibitory effects against viral replication, including compounds that modulate signal transduction pathways, protein processing, and DNA synthesis or repair; and (iii) the development of new chemical entities (NCEs) based on the structural and functional characteristics of the virus, including small molecules that target viral enzymes [56,57]. Generally, all of these approaches have been explored simultaneously to address the pandemic and can be empirically divided into virus-based and host-based approaches [56]. The immune evasion of SARS-CoV-2 infections involves the following mechanisms: (i) provoking a cytokine storm; (ii) blunting interferon responses; and (iii) suppressing antigen presentation by MHC class -I and class-II proteins. A better understanding of the immune evasion mechanisms that contribute to SARS-CoV-2 sustainability in the host will guide the rational, target-based design of efficient and specific immunomodulatory therapeutics [58]. Because the SARS-CoV-2 encoded nonstructural and accessory proteins play potential and critical roles in the immune evasion processes, targeting those proteins could be exploited to enrich drug discovery processes. Although any NSP could be considered a potential therapeutic target, the availability of crystal structures, the description of co-crystallized ligands, and their roles in viral pathogenicity could accelerate drug discovery [59]. Many studies have been performed to explore nonstructural and accessory proteins as potential therapeutic targets [60,61]. For example, drugs designed to target NSP-1 should aim to interact directly with the NSP-1 molecule or to target biological processes that are directly involved in the interactions between NSP-1 and host cells. Unlike the direct targeting of viral proteins, targeting downstream processes can diminish the potential emergence of mutational resistance, which could diminish the impacts of direct protein interactions, and likely increasing the success of identified therapeutic modalities [62]. Papain-like protease (PLpro), a domain found in NSP-3, represents an essential viral component that contributes to the downregulation of inflammatory and antiviral signaling processes. Accordingly, the modulation of PLpro activity could impair viral replication and, consequently, impede its role in host immune response evasion, making it a promising therapeutic target [63]. In addition, Shin et al. have reported that the lead compound GRL-0617 could inhibit SARS-CoV-2 PLpro, resulting in multiple therapeutic effects, including (i) viral pathogenicity inhibition; (ii) sustained antiviral interferon release; and (iii) reduced cellular viral replication. Therefore, targeting SARS-CoV-2 PLpro can suppress SARS-CoV-2 infection and improve antiviral immunity [64]. The macromolecular drug discovery approach to the development of SARS-CoV-2 PLpro inhibitors appears to have been more useful than the small-molecule drug discovery approach in terms of selectivity and potency. However, small-molecule inhibitors appear to be advantageous in terms of physicochemical properties and ADME (absorption, distribution, metabolism, and excretion) profiles [65]. The SARS-CoV-2 NSP-6 protein is known to interact with the sigma receptors involved in the regulation of the ER stress response and contribute to immune evasion through the inhibition of type 1 IFNs [48]. Small molecules targeting those receptors have been reported to inhibit virus replication and growth, blunting immune evasion events [66]. NSP-10 has been documented to play critical roles in the stimulation of the 3` -to-5` exoribonuclease and 2`-O-methyltransferase activities of NSP-14 and NSP-16; therefore, the targeting of NSP-10 could significantly blunt the immune evasion mechanism. However, little data is available regarding the structure of NSP-10, which limits the application of structure-based design to the development of potential leads [38]. Another important component in the immune evasion process is NSP-13 (helicase), which has multiple functionalities and interferes with IFN-α/β downstream signaling. The atomic structure of SARS-CoV-2 NSP-13 is currently unavailable, and no single published structural homolog has been identified that is suitable for virtual screening approaches [67]. Further studies remain necessary to develop new therapeutic strategies that are capable of targeting the SARS-CoV-2 accessory proteins involved in immune evasion. Given the urgency of the current pandemic and the limited available resources, repurposing existing drugs is an appropriate solution to the identification of a timely and effective therapy. Immune modulation has been demonstrated to be a useful strategy for the clinical management of previous viral outbreaks [59]. A growing body of literature has recognized the usefulness of immune-modulatory therapy for the management of severe clinical cases of COVID-19 [68,69].

### Glucocorticoids

Corticosteroids are synthetic steroidal hormones that were widely used to address the SARS and MERS outbreaks and are currently being applied to the management of COVID-19 [70]. Glucocorticoids represent an attractive therapeutic option for the management of ARDS-associated respiratory failure caused by the direct or indirect destruction of pulmonary tissues [64]. ARDS development involves a cascade of events that results in a massive inflammatory response and fluid accumulation in the alveolar space, which is a classic hallmark of ARDS [71]. Corticosteroid therapy can dampen the exaggerated pulmonary inflammatory response, inhibiting immune responses [72]. However, recently reported data has been insufficient to determine the effects of corticosteroid treatment on all-cause mortality or the duration of mechanical ventilation [73].

**FIGURE 3:**
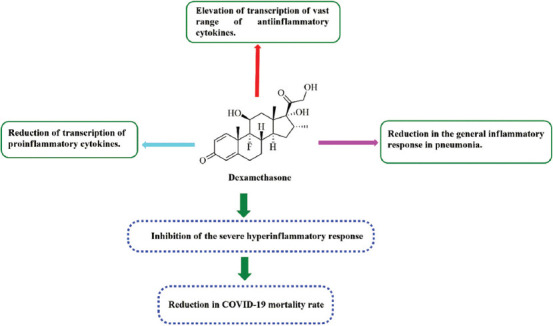
Mechanisms of dexamethasone as an immunomodulator in COVID-19, acting principally through three main pathways: diminishing the transcription of proinflammatory cytokines; promoting the transcription of anti-inflammatory cytokines; and dampening the general inflammatory response. All of these pathways contribute to the suppression of cytokine storm, eventually resulting in the reduction of disease mortality.

Limited data available in the literature has supported the efficacy of corticosteroid therapy in coronavirus infections [74]. Recently, the corticosteroid dexamethasone has been documented to reduce the mortality rate of COVID-19 patients by 20%–35% [75]. Dexamethasone appears to be 25-fold more potent than other corticosteroids, and this potency might justify its use in COVID-19 patients [70]. The immunomodulatory properties of dexamethasone are mediated by a cascade of reactions that involve both genomic and non-genomic mechanisms, resulting in the increased expression of anti-inflammatory molecules (such as IL-10 and annexin-1) and the decreased expression of proinflammatory cytokines (IL-2, IL-6, and TNF-α), eventually blunting the virus-induced cytokine storm (Figure 3) [76]. However, the inhibitory effects of dexamethasone on T and B cells, and the resulting immune repression, limit its use as a life-saving agent in severely ill COVID-19 patients [77]. Currently, dexamethasone is considered to be a front-line strategy that has been shown to decrease mortality in COVID19 patients. The currently updated World Health Organization (WHO) guidelines for COVID-19 drug therapy provide the following recommendations: (i) a strong recommendation for systemic corticosteroid therapy in severely or critically ill COVID-19 patients and (ii) a conditional recommendation against the use of systemic corticosteroid therapy in mild-to-moderate COVID-19 patients. The exact role played by corticosteroids in the immune modulation of COVID-19 remains unclear, and further clinical studies remain necessary to identify the specific immunomodulators [76].

### Anticancer drugs

The clinical course of tumorigenesis involves three primary manifestations: (i) hyperinflammatory responses; (ii) a dysfunctional immune system; and (iii) an Imbalanced coagulation state [78]. Similar disease manifestations have been identified in COVID-19 patients, offering a rationale for the potential application of anticancer drugs to the management of COVID-19 pathogenesis to improve overall clinical outcomes [78]. Several modes of action have been proposed for the pharmacological actions of anticancer therapies in COVID-19, including (i) the inhibition of structural protein translation (dactinomycin); (ii) the inhibition of the Janus kinase (JAK)-STAT pathway (ruxolitinib); and (iii) the blockade of the SARS-CoV-2 main protease (carfilzomib) [79]. However, these therapies represent double-edged swords that can slow viral clearance in addition to impairing humoral and cellular immunity, increasing the likelihood of secondary infections [80]. Therefore, immense attention should be paid to the clinical use of these therapies to avoid the oversuppression of the immune system and the development of subsequent complications among hospitalized COVID-19 individuals. Moreover, additional clinical trials remain necessary to support the usefulness of anticancer drugs for COVID-19 therapy [81].

### IL-6 inhibitors

IL-6 plays a significant role in the development of cytokine storm and is overexpressed during the severe inflammatory response associated with SARS-CoV-2 infection. IL-6 inhibitors, such as tocilizumab (monoclonal antibodies), inhibit the IL-6 transduction pathway and may represent a potential therapeutic choice for the COVID-19 associated with an exaggerated inflammatory response [82]. Current international guidelines suggest that IL-6 inhibitors might have therapeutic value if implemented in severely ill COVID-19 patients [83]. Clinical trials in multiple countries are currently ongoing to examine the use of the IL-6 inhibitors tocilizumab, siltuximab, and sarilumab in COVID-19 patients [69]. However, additional clinical studies are recommended to ensure the safety and efficacy of these potentially life-saving drugs [75].

### IL-1 inhibitors

Proinflammatory cytokines that belong to the IL-1 family have been reported to act as key regulators of IL-6 production [84]. Anakinra is a bio-engineered analog of the naturally occurring interleukin-1 receptor antagonist (IL-1ra) [85] that has been approved by the US FDA for the management of rheumatoid arthritis. Anakinra blocks IL-1β and IL-1a from binding to their receptors, resulting in diminished IL-1 activity [86]. However, the efficacy of anakinra for the treatment of COVID-19 has not yet been tested in controlled clinical trials [75]. Thus, its use should be limited to critical COVID-19 cases due to potential drug safety issues [85].

### Chloroquine and hydroxychloroquine

Chloroquine (CQ) is widely used in malaria treatment and prophylaxis. Its more water-soluble analog, hydroxychloroquine (HCQ), is primarily used due to its immunomodulatory properties [87]. HCQ has exhibited efficacy in lowering the COVID-19 mortality rate [88]. The immunomodulatory properties of HCQ have been ascribed to interference with lysosomal activity, resulting in the decreased expression of MHC-II and impaired antigen presentation, which subsequently reduces the release of a vast range of key proinflammatory cytokines [82]. Despite promising *in vitro* antiviral activity, no data support the efficacy of CQ and HCQ in the treatment of COVID-19 [89,90], and the latest largest international randomized controlled clinical trials, which were organized by the WHO to explore COVID-19 treatments, revealed no compensatory reductions in overall disease mortality following CQ or HCQ treatment [91].

### Intravenous immunoglobulins (IVIG)

Intravenous immunoglobulins (IVIG) are preparations comprised of a large pool of human antibodies, including a serum IgG fraction, which serves as a major component and consists principally of IgG1 and IgG2 subclasses, combined with smaller proportions of IgA, and IgM [92]. Initially, IVIG was developed as replacement therapy for cases of primary antibody deficiencies, and IVIG has been shown to exhibit modulatory effects on excessive immune system activation, involving both innate and adaptive immunity; therefore, IVIG has been used to treat a vast range of inflammatory-autoimmune disorders [93]. Several studies have reported the usefulness of IVIG therapy for the clinical management of various viral infections due to the effective reduction of accompanying exaggerated inflammatory responses [94,95]. IVIG has been documented to display beneficiary effects in patients with COVID-19. However, given the quality of available evidence, meaningful conclusions regarding the therapeutic efficacy of IVIG for the treatment of patients with COVID-19 cannot yet be drawn. Although the exact mode of action through which IVIG exerts effects in COVID-19 is not yet fully understood, the observed reduction in inflammatory mediators following IVIG administration in severely ill COVID-19 patients might explain the clinical value of this treatment. This anti-inflammatory effect could be attributed to various aspects, including (i) the inhibition of innate immune cells and effector T cell activation, (ii) complement cascade inhibition, and (iii) the expansion of the regulatory T cell (Treg) population [96]. The reported data indicate that passive viral neutralization may not be involved in the IVIG efficacy observed in COVID-19. Because IVIG contains antibodies capable of reacting with SARS-CoV-2 antigens, IVIG could inhibit superantigen-mediated T cell activation and cytokine release [97]. Passive virus neutralisation appears to be not responsible for the beneficial effects of IVIG.

### Serotherapy

Serum therapy describes passive antibody therapy, which has been successfully applied to the eradication and control of infectious diseases since the 19th century [98]. However, over the last five decades, substantial advancements in the discovery of potent antimicrobial drugs and effective vaccine development have significantly reduced the use of this strategy. Due to the limited therapeutic options available for COVID-19, the steadily increasing number of COVID-19-related deaths, the financial difficulties faced by some regions that may limit vaccine acquisition, and vaccine hesitancy, serotherapy may represent a viable therapeutic option for improving the clinical outcomes [99]. Despite limited data supporting the efficiency and safety of serotherapy for COVID-19 patients, evidence has indicated the potential usefulness of this strategy for critically ill patients [100,101]. The use of convalescent plasma rich in antibodies against SARS-CoV-2 has been suggested to neutralize the pathogen and provide passive immunomodulatory mediators, which assist the recipient in the control of excessive inflammatory cascades induced by the infectious agent [102,103]. Convalescent plasma shares similar modes of action as IVIG, including (i) direct virus neutralization, (ii) control of the hyperactive immune system (complement activation and cytokine storm), and (iii) immunomodulatory effects on the hypercoagulable state. Therefore, serotherapy might also demonstrate beneficial effects if administered to non-critically ill COVID-19 patients [103].

### Monoclonal antibodies (mAbs)

Monoclonal antibodies (mAbs; passive immunotherapy) have been used to prevent a wide range of infectious diseases and is considered to represent a new modality in the field of infectious diseases. Because mAbs are designed to target one specific substance in the human body, they are thought to provide efficient, safe, and specific interventional therapy compared with serotherapy [104]. Therefore, the inhibition of cytokine storm through the use of therapeutic mAbs might be effective for the COVID-19 associated ARDS [105]. During the COVID-19 pandemic, more than 50 clinical trials have been performed to examine the use of mAbs capable of targeting a vast range of cytokines; however, few have reached Phase III or IV [106]. The actions of mAbs are mediated by two key mechanisms: (i) a reduction in the viral load due to the inhibition of viral entrance into cells due to the binding of mAbs with either viral spike proteins or host cell receptors and (ii) the immunomodulatory properties that result in the suppression of uncontrolled and excessive immune responses by the host [106]. Because higher levels of IL-6 have been associated with disease severity and serve as an indicator of poor outcomes, most of the mAbs under clinical investigations are IL-6 inhibitors intended for use in moderate-to-severe COVID-19 patients [105]. Among existing mAb candidates, extensive clinical studies are ongoing for tocilizumab (Actemra^®^, Genentech), a recombinant monoclonal antibody that targets the IL-6 receptor and suppresses the signal transduction mechanisms thought to exacerbate cytokine storm [107,108]. Therefore, tocilizumab is expected to play a pivotal role in the future management of COVID-19-associated cytokine storm [105]. Other IL-6 inhibitors, including sarilumab, and siltuximab, are also being studied in clinical trials for their potential outcomes in the management of COVID-19 [109]. In addition, other mAbs are being investigated for possible effects on targets other than cytokines, which may be applied for prophylaxis and the management of COVID-19-related consequences [105].

### Anti-TNF therapy

An exaggerated inflammatory response is the primary driver of poor COVID-19 outcomes, and higher levels of TNF, a proinflammatory cytokine, have been linked to increased COVID-19 mortality [110]. Clinical observational data supports a potential therapeutic role for anti-TNF therapy in the treatment of COVID­19 [111], which could minimize the production of many proinflammatory cytokines, such as IL-1 and IL-6 [112]. The inhibition of cytokine production in COVID-19 patients might reduce the hyperinflammatory response and improve the clinical outcomes [110]. Further, anti-TNF therapy might reduce COVID-19-induced thrombosis, which could be attributed to the depletion of D-dimer and pro-thrombin fragments. Two off-patent anti-TNF agents, infliximab and adalimumab, are currently undergoing clinical trials for COVID-19 treatment.

In some patients, COVID-19 causes multiorgan failure; however, anti-TNF therapy has a wide margin of safety because it is eliminated from the body via the reticuloendothelial system, requiring no dose titration steps [110]. Secondary bacterial and fungal infections might be of concern during treatment with anti-TNF therapy; therefore, this treatment is contraindicated in individuals with latent tuberculosis [113,114].

### IFNs

**FIGURE 4:**
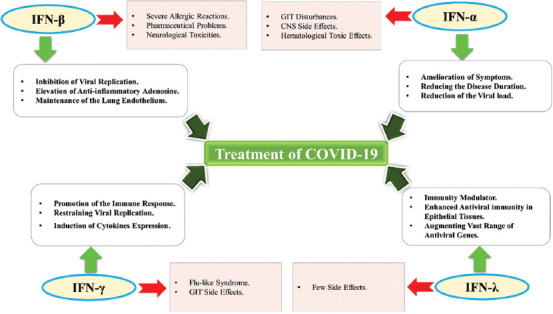
Immunotherapeutic effects and the potential side effects of IFNs in the context of COVID-19. IFN treatments all involve the inhibition of viral replication, the promotion of the host immune response, especially in epithelial tissues, and the elevation of anti-inflammatory cytokines.

The expression of IFN differs widely across coronavirus infections, with SARS-CoV-2 being less potent than other coronaviruses for the induction of IFN, signifying the pivotal role of IFN in COVID-19 (Figure 4). IFNs are protein-based, broad-spectrum, antiviral, and anti-inflammatory cytokines that interact with cell surface receptors to stimulate the JAK-STAT signaling pathway, which ultimately leads to the production of the antiviral enzyme RNase L and the proinflammatory chemokine CXCL10 [68]. The proinflammatory responses mediated by type I and II IFNs are induced by stimulating the expression of a vast array of immune cell genes. The activated immune cells kill infected cells or deactivate the viruses. Therefore, the maintenance of a satisfactory level of the antiviral type I IFNs is essential to sustaining the innate and adaptive human immune response [115]. Studies have revealed that IFN-a could reduce viral load during the early stages of SARS-CoV-2 entry, contributing to symptom relief and shortening disease duration [116].

IFN-β-1a has been reported to be effective and safe for the management of a variety of viral diseases, such as SARS-CoV. Due to the similarities between SARS-CoV and SARS-CoV-2, IFN-β-1a was added to the antiviral therapy against COVID-19 [68]. A recent study demonstrated the ability of IFN-β-1b to restrain the replication of SARS-CoV-2, which also resulted in its inclusion in antiviral combination therapies [117]. IFN-β-1a and -1b have been reported to be highly effective in COVID-19 patients [118,119], which may be partially attributed to their positive roles in lung tissues, where they are involved in endothelium maintenance and the elevation of anti-inflammatory adenosine levels [120].

The IFN-γ (type II IFN)-mediated stimulation of gene transcription has been shown to eventually inhibit viral multiplication. Moreover, an improvement in the overall immune response has been documented due to the ability of IFN-γ to stimulate MHC class II receptors [68]. IFN-γ demonstrates the weakest ISG response but could promote the surface expression of ACE2 [121]. The safety of IFN-γ treatment has previously been demonstrated, but its effectiveness for COVID-19 treatment remains limited and requires confirmation. IFN-λ is a type III IFN that stimulates epithelial cells and reduces the macrophage-mediated activity of IFN-a and -b [68]. Currently, pegylated IFN-λ1 (peg-IFN-λ1) is the only therapeutic form available on the market [122]. IFN-λ exhibited an enhanced response against SARS-CoV-2 when tested *in vitro*, and it exerts its therapeutic effect primarily through the inhibition of viral infection via the promotion of antiviral immunity; after infection, IFN-λ can also reduce viral production and diffusion [122,123]. The most important characteristic of IFN-λ in the context of COVID-19 is the general ack of proinflammatory activity in the lungs compared with that induced by type I IFNs. Though IFN-λ might be more efficacious than type I IFNs for COVID-19 therapy, additional studies remain necessary to assess its safety profile [68]. Unlike type I IFNs, which are widely used in clinical settings, type III IFNs have yet to be approved for therapeutic applications. However, the distinctive properties of the type III IFN response, such as (i) specific, (ii) long-lasting, and (iii) non-proinflammatory effects, indicate that IFN-λ may represent an attractive therapeutic option for COVID-19 [124].

Reductions in the viral load and clinical symptoms of COVID-19 have been achieved by the early use of IFNs [68]. In a clinical trial examining 446 COVID-19 patients, the administration of IFN-a2b during the early phase of COVID-19 resulted in promising clinical outcomes. By contrast, IFN-a2b administration during late-stage COVID-19 has been shown to increase mortality [125]. Clinically silent neutralizing autoantibodies against type I IFNs are present in at least 10% of critically ill COVID-19 patients and might contribute to their therapeutic failure [126].

## CONCLUSION

The COVID-19 situation has worsened during the second wave in many countries, particularly in low-income countries. Several studies have reported that the severity of COVID-19 was associated with a reduced type I IFN response and the increased production of proinflammatory mediators. Therefore, understanding the molecular interactions between SARS-CoV-2 and the host defense machinery is crucial for improving treatment interventions and the development of an effective vaccine. SARS-CoV-2 NSPs and accessory proteins can engage in the strategies that result in the modulation of the host immune response, depending primarily on the blockade of type I IFN production and the ensuing signaling pathway. Some NSPs, including PLpro and NSP-10, and accessory proteins, including ORF3a and ORF9c, may promote inflammatory reactions. Immunomodulatory therapy has been successfully applied to the treatment of COVID-19 patients, which further indicates the critical role of immune evasion as a key regulator of morbidity and mortality associated with this disease. Further studies remain necessary to explore the role played by SARS-CoV-2 NSPs and accessory proteins in the regulation of proinflammatory mediators. Additional study remains necessary to establish the best approaches to block the ability of SARS-CoV-2 to hijack the type I IFN response.
